# Peripheral nerve injury is accompanied by chronic transcriptome-wide changes in the mouse prefrontal cortex

**DOI:** 10.1186/1744-8069-9-21

**Published:** 2013-04-18

**Authors:** Sebastian Alvarado, Maral Tajerian, Magali Millecamps, Mathew Suderman, Laura S Stone, Moshe Szyf

**Affiliations:** 1 Department of Pharmacology and Therapeutics, McGill University, Faculty of Medicine, 3655 Promenade Sir William Osler, Montréal, Québec H3G 1Y6, Canada; 2 Sackler Program for Epigenetics & Developmental Psychobiology, McGill University, 3655 Promenade Sir William Osler, Montréal, Québec H3G 1Y6, Canada; 3 Department of Neurology and Neurosurgery, McGill University, Faculty of Medicine, 3801 University Street, Montreal, Quebec H3A 2B4, Canada; 4 Alan Edwards Centre for Research on Pain, McGill University, 740 Dr. Penfield Avenue, Montreal, Quebec H3A 1A4, Canada; 5 Faculty of Dentistry, McGill University, 3640 University Street, Montreal, Quebec H3A 2B2, Canada; 6 Department of Anesthesiology, Anesthesia Research Unit, McGill University, Faculty of Medicine, 3655 Promenade Sir William Osler, Montreal, Quebec H3G 1Y6, Canada

**Keywords:** Neuropathic pain, Chronic pain, RNA sequencing, Transcriptome, Noncoding RNA, Neuronal plasticity

## Abstract

**Background:**

Peripheral nerve injury can have long-term consequences including pain-related manifestations, such as hypersensitivity to cutaneous stimuli, as well as affective and cognitive disturbances, suggesting the involvement of supraspinal mechanisms. Changes in brain structure and cortical function associated with many chronic pain conditions have been reported in the prefrontal cortex (PFC). The PFC is implicated in pain-related co-morbidities such as depression, anxiety and impaired emotional decision-making ability. We recently reported that this region is subject to significant epigenetic reprogramming following peripheral nerve injury, and normalization of pain-related structural, functional and epigenetic abnormalities in the PFC are all associated with effective pain reduction.

In this study, we used the Spared Nerve Injury (SNI) model of neuropathic pain to test the hypothesis that peripheral nerve injury triggers persistent long-lasting changes in gene expression in the PFC, which alter functional gene networks, thus providing a possible explanation for chronic pain associated behaviors.

**Results:**

SNI or sham surgery where performed in male CD1 mice at three months of age. Six months after injury, we performed transcriptome-wide sequencing (RNAseq), which revealed 1147 differentially regulated transcripts in the PFC in nerve-injured vs. control mice. Changes in gene expression occurred across a number of functional gene clusters encoding cardinal biological processes as revealed by Ingenuity Pathway Analysis. Significantly altered biological processes included neurological disease, skeletal muscular disorders, behavior, and psychological disorders. Several of the changes detected by RNAseq were validated by RT-QPCR and included transcripts with known roles in chronic pain and/or neuronal plasticity including the NMDA receptor (glutamate receptor, ionotropic, NMDA; *grin1*)*,* neurite outgrowth *(*roundabout 3*; robo3),* gliosis *(*glial fibrillary acidic protein*; gfap),* vesicular release (synaptotagmin 2*; syt2),* and neuronal excitability *(*voltage-gated sodium channel, type I*; scn1a)*.

**Conclusions:**

This study used an unbiased approach to document long-term alterations in gene expression in the brain following peripheral nerve injury. We propose that these changes are maintained as a memory of an insult that is temporally and spatially distant from the initial injury.

## Background

Peripheral nerve injury can result in a multitude of changes within an organism, including motor dysfunction, pain and associated cognitive and emotional comorbidities. While acute pain related to an injury is protective and normally resolves, chronic pain can be detrimental to the overall wellbeing and functioning of the individual. Indeed, occasionally the “memory” of injury, in the form of chronic pain, persists long after the initial recovery phase and becomes difficult to reverse. This is due, in part, to changes in anatomy and function that take place in the peripheral as well as the central nervous system. These changes can occur at many levels: individual molecules, synapses, cellular function, and network activity [1]. It is therefore not surprising that injury is often accompanied by local or systemic alterations in gene expression. To date, several reports have identified strong links between injury and transcriptional changes in the peripheral nervous system [2], blood [3] and in the brain [4]. However, the full profile of transcriptional changes that accompany chronic pain in response to peripheral injury is unknown.

The fact that peripheral injury results in chronic behavioral changes suggests that transient exposure to injury is chronically embedded in the transcription programming within the central nervous system, resulting in altered phenotypic behaviors. In the current study, we tested this hypothesis by focusing on the transcriptional changes that occur in the prefrontal cortex (PFC) of chronically neuropathic mice six months after the induction of neuropathy. This area was chosen based on evidence indicating its involvement in pain modulation [5,6] and epigenetic differences that accompany peripheral nerve injury [7]. In humans with back pain, reversible pathological changes in both cortical thickness and functional activation have been shown in the PFC [8]. In animal models of neuropathic pain, the PFC undergoes synaptic re-organization as early as 8 days following nerve injury [9], and shows reductions in grey matter 5 months post-injury [10]. Since the PFC has also been implicated in depression and anxiety [11], common co-morbidities of chronic pain, transcriptional changes in brain regions such as the PFC can also provide an explanation for these co-morbidities.

While brain-specific transcription associated with neuropathic pain and injury has been previously investigated, few studies have looked at the effects at time points longer than ~ 1 month post-injury, whereas individual patients often suffer for many years following the initial injury. This distinction is critical as the development of co-morbid conditions, such as anxiety-like behavior in the rat, takes multiple months to develop [10]. Thus, early time points might not fully incorporate the impact of long term chronic pain on CNS plasticity. Furthermore, previous studies have been limited by microarray-based technologies that are inherently biased by probe density/design and limited to coding mRNA transcripts [12]. We therefore investigated the long-term (6 months post-injury) transcriptional changes induced by peripheral injury in the prefrontal cortex using whole transcriptome sequencing (RNAseq) in a mouse model of chronic pain (Spared Nerve Injury, SNI). Furthermore, we employed bioinformatic tools to identify functional gene clusters that were altered in the prefrontal cortex.

The aim of this work is to provide a comprehensive and unbiased look at the molecular correlates of peripheral nerve injury and catalog long-term transcriptional changes that may play a role in the pathologies associated with injury and pain. We believe that our findings will help shed light on the “signature” of painful neuropathy in the brain at both the molecular and network levels.

## Results

### Peripheral injury is accompanied by behavioral signs of neuropathic pain six months post-injury

The persistence of nerve injury-induced hypersensitivity to mechanical and cold stimuli and injury-related motor impairment were confirmed six months following SNI (data not shown; mechanical thresholds (grams) = 0.20±0.05 in SNI vs 0.82±0.07 in controls, p<0.0001; acetone-evoked behaviors (seconds) = 2.9±0.4 vs. 0.4±0.05 in controls, p>0.0001; motor impairment (latency to fall from accelerating rotarod) = 76±11 in SNI vs. 225±21 in controls, p>0.0001; n=10/group).

### Peripheral injury is accompanied transcriptomic changes in the prefrontal cortex six months post-injury

**Figure 1:**
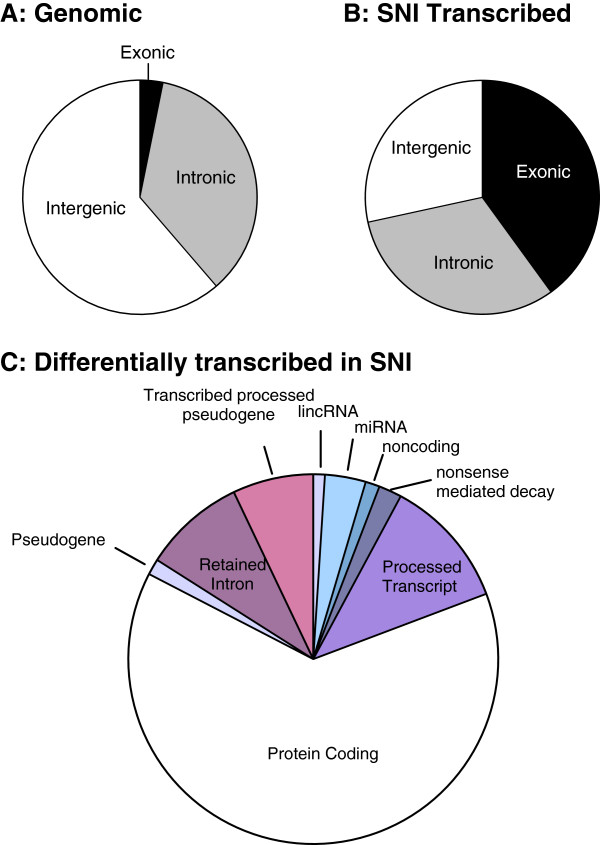
**Direction and nature of transcriptome expression alterations in the prefrontal cortex six months after injury in SNI and Sham animals.** (**A**) Distribution of exonic, intergenic and intronic elements within the mouse genome. (**B**) Distribution of transcribed exonic, intergenic and intronic elements in SNI PFC transcriptome relative to Sham PFC. (**C**) Distribution of differentially expressed RNAs in the PFC associated with peripheral nerve injury.

Six months following SNI, significant changes in the expression levels of 1147 different transcripts were identified in the prefrontal cortex (Figure 1, Additional file 1: Table S1). Considering the genomic base pair representation of exonic (3.2%), intronic (35.5%) and intergenic (61.3%) elements in mouse (Figure 1A), the PFC SNI-associated transcriptome was equally partitioned between coding RNA accounting for 40% (exonic) and 60% non-coding (intergenic+intronic) base pairs of the transcriptome (Figure 1B). Within differentially expressed transcripts in SNI vs. sham control animals, the largest transcriptional changes were observed in protein-coding exons (63.4%) with the rest made up of non-coding RNA. Within non-coding RNAs, the largest observed changes were in non-translated transcripts (retained introns and processed transcripts) followed by classes of microRNAs, transcribed processed pseudogenes, lincRNAs and nonsense mediated decay (Figure 1C).

The transcripts with the largest log_2_fold changes in RNA expression in response to SNI (summarized in Table 1) included several transcripts with previously uncharacterized roles in the brain. Within this subset, specific transcripts with a role in subcellular structure (*actbl*, *col11a2*, *krt12*, *krt20*) and development pathways *(lrat*[13], *aldh3a1*[14], *crb1*[15]) show some of the greatest transcriptional changes. These transcripts showed large transcriptional differences from control ranging between 4.20 and 5.20 log_2_fold change for repressed genes and between 2.61 and 5.60 log_2_fold for induced genes.

**Table 1:** Top upregulated and downregulated transcripts in SNI

**Gene**	**Chr**	**Start**	**End**	**p-Value**	**Log**_**2**_**fold change**
**Brain-specific angiogenesis inhibitor 1-associated protein 2-like protein 1**	**5**	145036642	145036834	1.23E-08	**−5.20**
**A330050B17Rik**	**3**	35921194	35924028	5.37E-07	**−4.92**
**Actin, beta-like 2**	**13**	112045221	112047957	8.12E-10	**−4.56**
**mmu-mir-220**	**6**	136348946	136349031	5.54E-20	**−4.53**
**Crumbs homolog 1**	**1**	141142194	141145652	4.49E-5	**−4.43**
**Collagen, type XI, alpha 2**	**17**	34189110	34189163	2.34E-4	**−4.23**
**Thyrotropin-releasing hormone receptor**	**15**	44065008	44065236	2.34E-4	**−4.22**
**Gm14204**	**2**	158421208	158422248	1.10E-12	**−4.08**
**Gap junction alpha-5**	**3**	96879570	96881339	2.37E-11	**5.60**
Keratin 20*	**11**	99299001	99299464	7.97E-6	**4.65**
**Mediator complex subunit 23**	**10**	24589792	24589952	4.30E-4	**4.08**
**Keratin 12**	**11**	99280858	99281014	3.83E-10	**4.08**
**40S ribosomal protein S28**	**17**	33959981	33960061	1.47E-13	**3.76**
**A830036E02Rik**	**11**	99283179	99283574	1.77E-17	**3.69**
**Lecithin-retinol acyltransferase**	**3**	82696501	82701050	4.22E-08	**3.10**
**Thiosulfate sulfurtransferase (rhodanese)-like domain containing 2**	**4**	46151364	46151566	5.24E-4	**2.77**
**Complement component 1 r subcomponent A**	**6**	124463670	124463895	5.24E-4	**2.73**
**Aldehyde dehydrogenase 3 family member A1**	**11**	61028052	61028260	3.22E-6	**2.61**

Changes are labeled as log_2_fold change.

**Figure 2:**
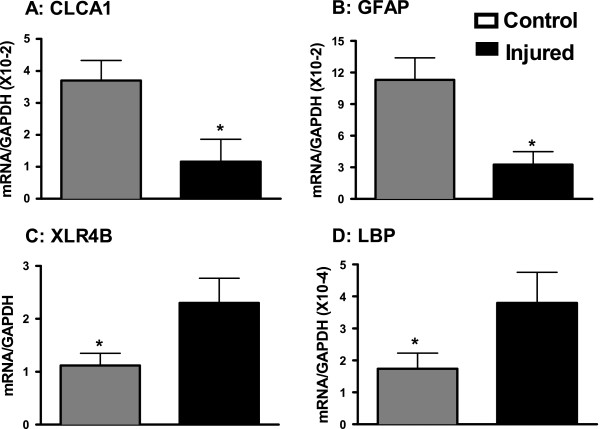
**Validation of transcript mRNA expression.** Quantitative PCR validation of downregulated transcripts CLCA1 (**A**) and GFAP (**B**) and upregulated transcripts XLR4B (**C**) and KRT20 (**D**) relative to GAPDH. *=p<0.05. n=8/group. Error bars = S.E.M.

**Figure 3:**
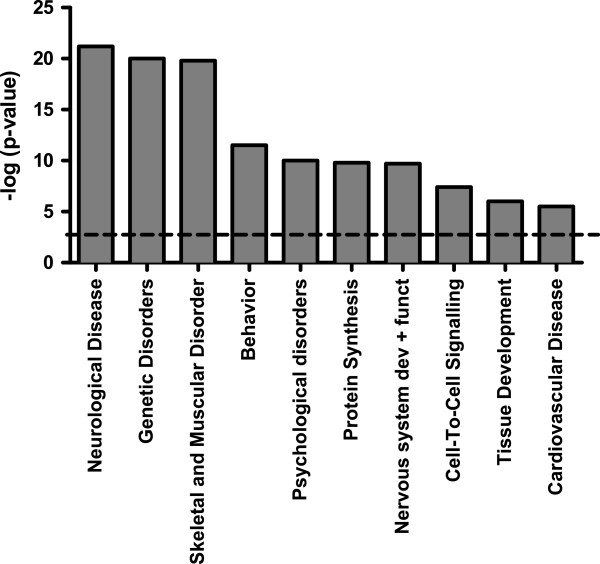
**Functional pathway analysis.** Nerve injury affects transcriptional programs unique to neurological disease, skeletal and muscular disorders, psychological disorders and changes in behavior scored and ranked according to Ingenuity Pathway Analysis using Fisher’s exact test. The dotted line indicates the threshold value of p<0.05.

**Figure 4:**
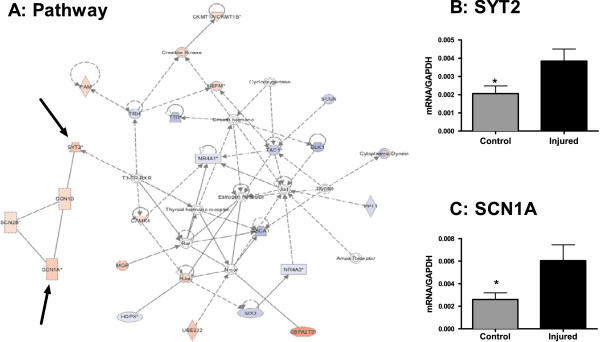
**Cellular growth and differentiation.** Nerve injury results in distinct changes in transcription in pathways involved in cellular growth and proliferation. (**A**) RNAseq and IPA identified interacting networks affecting cell cycle, cell proliferation and cellular development. Up-regulated transcripts are marked with red while downregulated transcripts are marked in blue. SYT2 (**B**) SCN1A (**C**) transcripts were validated by qPCR. *=p<0.05. n=8/group. Error bars = S.E.M.

**Figure 5:**
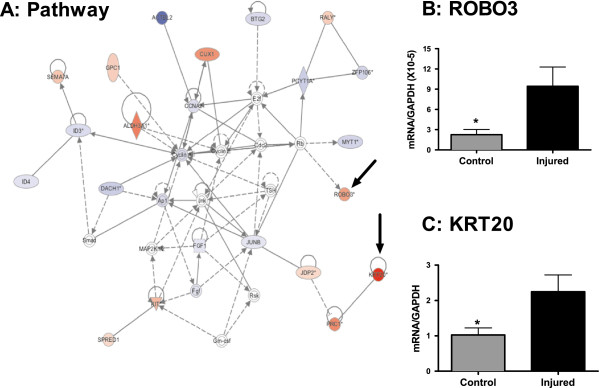
**Cell cycle and growth.** SNI causes distinct changes in transcription in pathways involved in cell cycle and growth. (**A**) RNA sequencing and IPA identified interacting networks affecting cell cycle, cellular growth and proliferation and cellular development. Upregulated transcripts are marked with red while downregulated transcripts are marked in blue. ROBO3 (**B**) and KRT20 (**C**) transcripts were validated by qPCR. *=p<0.05. n=8/group. Error bars = S.E.M.

**Figure 6:**
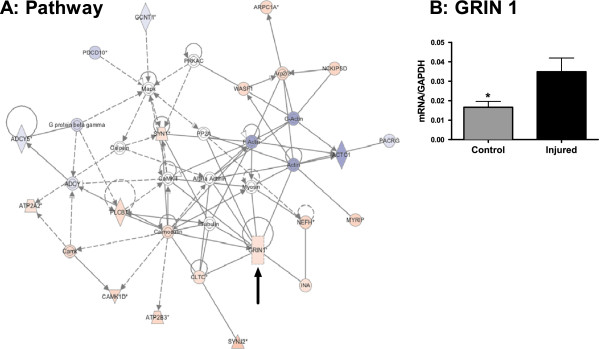
**Neuronal development.** SNI causes distinct changes in transcription in pathways involved neuronal development. (**A**) RNA sequencing and IPA identified interacting networks affecting cellular assembly and organization and nervous system development and function. Upregulated transcripts are marked with red while downregulated transcripts are marked in blue. (**B**) GRIN1 transcript was validated by qPCR. *=p<0.05. n=8/group. Error bars = S.E.M.

We further classified differentially expressed transcripts in SNI animals that have brain-specific functions (Summarized in Table 2). Examples include down-regulation of neurotransmitter channel and receptor subunits *gabrg1/3, clca1, slc14a1* and *drd2* and the astrocyte marker *gfap* while sodium channel subunit *scn1a*, NMDA subunit *grin1*, and promoters of neuronal growth *xlr4b, robo3, prc*, and *cux1* were all up-regulated in PFC from injured mice. Specific transcripts identified with asterisks in Tables 1 and 2 were further validated with RT-qPCR (labeled with asterisks) and summarized in Figures 2A-D, 3B-C, 4B-C and 5B. Within these validated genes, *robo3, scn1a, grin1, xlr4b, krt20, syt2,* and *lbp* showed marked induction following SNI while *gfap* and *clca1* showed marked repression (unpaired 2-tailed t-test, p<0.05, n=8). Whereas the genes in Figure 2 were selected for validation due to an interesting role in the CNS, Figures 4, 5, and 6 highlight validated genes within the context of an identified functional gene cluster.

**Table 2:** Biased upregulated and downregulated transcripts in SNI with known brain-specific functions

**Gene**	**Chr**	**Start**	**End**	**p-Value**	**Log**_**2**_**fold change**
Calcium-activated chloride channel regulator 1*	**3**	144409728	144409992	4.95E-04	**−3.19**
**Solute carrier family 14 (urea transporter), member 1**	**18**	78308093	78308285	2.90E-06	**−2.37**
**Gamma-aminobutyric acid receptor subunit gamma-1**	**5**	71185885	71185952	8.90E-05	**−2.17**
**Activating transcription factor 3**	**1**	192994175	192995558	8.97E-07	**−1.98**
**Fatty acid binding protein 7**	**10**	57505312	57505484	4.36E-12	**−1.44**
**Gamma-aminobutyric acid receptor subunit gamma-3**	**7**	64433752	64435107	3.29E-04	**−1.38**
Glial fibrillary acidic protein *	**11**	102753225	102753268	2.40E-04	**−1.24**
**Fos**	**12**	86816542	86816649	4.62E-04	**−0.90**
**Dopamine receptor D2**	**9**	49215006	49216282	1.44E-05	**−0.88**
X-linked lymphocyte-regulated 4B *	**X**	70463702	70467280	1.72E-03	**3.87**
Lipopolysaccharide-binding protein*	**2**	158139209	158139364	3.31E-04	**2.54**
**Protein regulator of cytokinesis 1**	**7**	87460000	87461144	5.70E-05	**2.40**
**Cut-like homeobox 1**	**5**	137041250	137041567	1.46E-04	**2.24**
Roundabout, axon guidance receptor, homolog 3*	**9**	37227254	37227393	1.51E-05	**1.96**
Sodium channel, voltage-gated, type I, alpha subunit*	**2**	66166462	66166603	4.34E-04	**1.10**
Synaptotagmin-2*	**1**	136644052	136649726	9.10E-09	**0.91**
**Synapsin 1**	**X**	20497539	20498130	1.72E-03	**0.59**
Glutamate [NMDA] receptor subunit zeta-1*	**2**	25174149	25174615	3.31E-04	**0.57**

Changes are labeled as a log_2_fold change.

### Peripheral nerve injury results in changed brain-specific transcriptional programs

Ingenuity Pathway Analysis (IPA) identified specific networks that were dysregulated six months post-SNI in the prefrontal cortex. Biological functions with a cutoff p-value < 0.05 were considered statistically significant. We identified the following biological functions specific to peripheral nerve injury: neurological disease (p-value= 2.02E-21-1.46E-02), behavior (p-value= 2.68E-12-1.31E-02), psychological disorder (p-value=7.80E-11- 1.46E-02), protein synthesis (p-value=1.70E-10 to 4.23E-03), and nervous system development and function (p-value=5.93E-10 to 1.42E-02) (Figure 6, Additional file 2: Table S2). In our screen, genetic disorders and skeletal muscular disorders emerged but due to an unclear role in CNS we did not perform further validation within these pathways.

With each biological function comprised of hundreds of functional gene clusters we then focused on transcriptional clusters previously associated with neurological function. Within this scope, peripheral injury resulted in up-regulation of pathways involved in cellular growth and proliferation (Figure 3A), molecular transport and neurological disease (Figure 4A) and neuronal development (Figure 5A). We validated the induction of representative genes included in these clusters by QRT-PCR: *robo3* and *krt20* in the cellular growth and proliferation pathway (Figure 3B-C), *scn1a* and *syt2* in the molecular transport and neurological disease pathway (Figure 4B-C) and *grin1* in the neuronal development pathway (Figure 5B).

## Discussion

Our results delineate for the first time a transcriptomic signature in the prefrontal cortex resulting from peripheral injury six months prior. Interestingly, both coding and non-coding transcripts are altered.

The coding transcripts include both genes that were previously implicated in the pathology associated with neuronal plasticity as well as genes with yet an unknown role in brain function, neuronal plasticity or in chronic pain. We further mapped the functional gene pathways whose transcription was altered, identified specific clusters involved in neuronal plasticity and validated candidate genes within these pathways. Genes known to play a role in brain structure and function that were differentially expressed and validated in response to peripheral injury were: *clca1*, *syt2, grin1, scn1a, krt20, xlr4b, gfap, lbp* and *robo3*. Considering the importance of the PFC to chronic pain and its associated co-morbidities, these broad, functionally-relevant changes in the transcriptome of the PFC provides a possible substrate for the long-term systemic effects of peripheral injury and may elucidate transcriptional mechanisms of supraspinal pathologies associated with chronic pain.

### Coding/noncoding transcriptomic changes

While annotated coding transcripts account for about a third (n=209000) of the long-term differential transcriptome in response to SNI, the remaining differentially-expressed transcripts were non-coding unannotated RNA (intergenic+intronic) (n=435000) (Figure 1B). Within differential exonic transcripts, we further classified all annotated genes into their respective RNA classes (Figure 1C). Approximately 60% of exonic elements represent protein-coding genes and the remaining 40% demonstrate a wide array of noncoding RNA with a previously uncharacterized role in peripheral nerve injury (Additional file 1: Table S1). The role of noncoding RNA has been discussed extensively [16,17] and it is known to be involved in a variety of functions ranging from translation [18], splicing [19] to transcriptional regulation [20]. The fact that SNI induces non-coding (both annotated and un-annotated) transcripts in the brain that could be detected long after the initial injury suggests that they might be playing a functional role in the brain response to peripheral injury. However, the mechanisms involved remain unknown at this stage. Nevertheless, our data provide further support to the emerging idea that genome-function in the brain involves more than the commonly studied protein coding gene sequences.

Annotated SNI-associated transcriptional differences were categorized into biological functions using ingenuity pathway analysis and we identified statistically significant affected gene pathways pertinent to neurological disease, behavior and psychological disorders (Figure 6 Additional file 2: Table S2). The SNI-associated transcriptome of the PFC appears particularly relevant to several of the co-morbidities associated with peripheral nerve injury such as poor sleep, anxiety and depression [21] as the PFC is highly implicated in these conditions [22-24]. Considering the identified molecular pathways (Figure 6), chronic neuropathic pain may alter higher order biological functions that may mediate these behavioral disorders.

### Genes involved in chronic injury and altered neuronal growth and proliferation in the PFC

The up-regulation of *robo3, krt20, xlr4b* in response to peripheral injury and overall pathway analysis suggests altered regulation of gene networks involved in neuronal cellular growth (Figures 2 &3, Additional file 2: Table S2). Previous work using the same model of peripheral nerve injury reported altered neuronal growth in injured animals characterized by longer basal dendrites with more branches in cortical layer 2/3 pyramidal neurons in injured vs. control animals [9]. The cell adhesion membrane protein ROBO3, which is up-regulated in the injured mice, has been shown to be essential for axonal guidance in drosophila [25] and is a regulator of cortical interneuron growth in mice [26] and serotonergic neuronal differentiation [27]. While not previously considered in the context of peripheral injury, its role in regulating morphology and function of neurons is consistent with a possible contribution to cortical remodeling in response to injury (Figure 3B).

*xlr4b*, a non-coding RNA, has been shown to regulate chromatin remodeling as well as modulate cognitive defects in a mouse model of Turner’s Syndrome [28]. Upstream regulators of *xlr4b*, *cux1* and *cux2* control the number and maturity of dendritic spines in cortical neurons in cortical layer 2/3 pyramidal neurons [29]. Interestingly, in addition to *xlr4b, cux1* is up-regulated (4 fold) in the SNI animals, supporting the hypothesis that this gene regulatory pathway is activated (Additional file 1: Table S1 and Figure 3A). *clca1,* which is down-regulated in the PFC in response to peripheral nerve injury (Figure 2A), encodes a calcium chloride channel. Calcium chloride channels are highly conserved structurally but have diverse expression patterns and physiological functions [30,31]. CLCA1 has been implicated in neuronal death [32], suggesting that CLCA1 might play role in neuronal reorganization in the PFC.

Lastly, while *krt20* is not directly implicated in neuronal growth, as a soft keratin and intermediary filament, its expression is ubiquitous. *krt20* is up-regulated in the PFC (Figure 3C) in response to peripheral nerve injury and it is considered a member of functional pathways involved in cell cycle and proliferation (IPA analysis). Taken together, these data are consistent with alterations in regulation of cell growth and proliferation as a chronic response to peripheral nerve injury. However, direct experiments need to test whether these transcriptional changes in the PFC are causally involved in altering neuronal or associated cell growth in the PFC.

### Molecular transport and neurological disease

Chronic pain changes the brain at various levels: changes in cortical grey matter [33,34], white matter [35], and overall cortical excitability [36] have all been reported. Recent studies have shown that chronic pain is linked to changes in synaptic structure and function in the primary somatosensory cortex [37], hippocampus [38], and anterior cingulate cortex [39]. It is therefore not surprising that several neurological disorders are co-morbid with chronic pain. Previous reports have shown chronic pain is associated with impaired decision-making [40], anxiety, depression and sleep disorders [41]. In the SNI model, the PFC exhibits signs of damage following peripheral injury [9,10]. Considering that the PFC is shown to regulate higher order emotional processing, transcriptional changes in the PFC can identify putative candidates mediating known co-morbid pathologies seen with chronic injury and/or pain.

Mutations in sodium channels have been previously implicated in pain and in the modulation of neuropathic and inflammatory pain [42,43]. In the present study, IPA-identified pathways reveal that the gene encoding sodium channel *scn1a,* which is a member of the neurological disease functional gene cluster, is up-regulated in the SNI group (Figure 4C). Familial missense mutations in *scn1a* have been linked to epilepsy [44], migraines [45] and brain structure in aging individuals [46]. Interestingly, these familial missense mutations can result in increased sodium channel activity; for example, the D188V mutation (linked to epilepsy) results in impaired inactivation, and the Q1489K mutation (linked to familial hemiplegic migraine) results in changes in channel gating which include accelerated recovery from inactivation and increased persistent current [47]. Thus, the functional result of these missense mutations may be analogous to the impact of nerve injury-induced up-regulation of the same channel.

This is the first demonstration of long-term overexpression of *scn1a* in the prefrontal cortex following peripheral injury. Nevertheless our data is consistent with previous reports showing up-regulation of SCN3A, for example, in dorsal root ganglia following axotomy [48]. The data is consistent with the hypothesis that *scn1a* over-expression (and to a lesser degree *scn1b, scn2b*, and *scn4b* (Additional file 1: Table S1)) is involved in increased cortical excitability [49] associated with chronic pain.

*Synaptotagmin II* (*syt2*) is up-regulated in our model in response to peripheral injury (Figure 3B) which is consistent with its role in neurological function and disease. Synatoptoagmins are Ca2+ sensors involved in vesicular trafficking and exocytosis and mediate vesicular release important for neurotransmission [50]. Syt2 was previously reported to be involved in vesicular GABAergic transmission [51], and may therefore also be involved in pain-mediated alterations in GABA and glutamate receptor activation known to accompany models of peripheral injury [52].

LBP, a mediator of innate immunity [53], is up-regulated in the PFC of animals with peripheral nerve injury. Interestingly, LBP-KO mice show increased spine density and abnormal spine morphology, indicating a role for LBP role in synaptic pruning [54]. We speculate that its role in TLR4 signaling may link it to the neuroinflammation and depression induced by treatment with lipopolysaccharide [55]. Altered LBP expression in the PFC in response to SNI might be playing similar roles in the gene networks, possibly mediating cortical neuroinflammation in animals with peripheral nerve injury.

### Nervous system development and function

One of the functional gene pathways that is highly enriched by genes that are differentially expressed six months after nerve injury is the pathway involved in nervous system development and functions (Figure 5, Additional file 2: Table S2). One member of this cluster which is up-regulated by SNI is *grin1* (NMDA receptor subunit 1). NMDA receptors are linked to neuroplastic changes including long-term potentiation [56] and implicated in psychiatric disorders [57-59] and anxiety [60,61].

Within the nervous system development and function cluster we found a marked decrease in the expression of *gfap*, a marker for astrocytes. Astrocytes are implicated in synapse maintenance, secretion of neurotrophins, and overall function in maintaining neuroplasticity. Previous work has shown that peripheral nerve injury is associated with increased levels of *gfap* in the spinal and medullary dorsal horns [62,63], anterior cingulate cortex [64-66], and the periaqueductal gray, during early and intermediate time points after injury [65]. However, there is evidence showing *gfap* is down-regulated in the PFC in psychological disorders [67,68]. Furthermore, pain-related pathological changes in neuroinflammation in the spinal cord, such as microglial activation, may occur in the absence of parallel dysregulation in supraspinal structures [69], and glial activation following spinal cord injury is both spatially and temporally regulated. Thus, the well documented increases in astroctye activation in the spinal cord following nerve injury [70] may not be mirrored in the PFC and/or *gfap* could be up-regulated during the early and middle stages of injury but down-regulated at very chronic stages once the co-morbidities have appeared.

## Conclusions

We demonstrate broad changes in gene expression in the mouse prefrontal cortex six months after peripheral nerve injury, illustrating a long-term impact of a peripheral injury on brain genome function. The use of RNAseq allowed for an unbiased picture of the transcriptomic changes involved in chronic neuropathy in the PFC. Furthermore, the ingenuity pathway analysis revealed functional gene networks that were significantly implicated including networks involved in brain development and function. These reported changes and their functional analysis will generate hypotheses on the molecular mechanisms that mediate chronic pain and its co-morbidities that will need to be tested in future experiments.

These wide-spread changes in gene expression in the PFC are consistent with our previous report demonstrated significant genome-wide changes in global DNA methyation that we predicted would result in the dysregulation of hundreds of genes. Thus, epigenetic mechanisms that embed the transient peripheral injury into a long-term programmatic change in gene function in the brain may be contribution to these mechanisms [7].

Although we demonstrate a causal relationship between peripheral injury and a transcriptome change six months later, it is unknown whether these are the same changes that occurred in the brain at the time of injury or whether (and more probably) a cascade of gene expression alterations led to the chronic profile observed in our study. Furthermore, it is unknown if similar changes occur in other spinal or supraspinal regions. Further experiments are required at multiple time point and in additional brain regions, including those not implicated in pain signaling, before the significance of these findings can be fully understood. Finally, there is currently no evidence that these patterns are the cause of chronic pain or its associated co-morbidities. Nevertheless, our study strongly supports the plausibility that long-term changes in gene expression in the CNS are involved in chronic pain and its associated behaviors, and generates hypotheses on the genes and functional gene networks that might be involved.

## Methods

### Animals

8–10 week-old male CD1 mice (Charles River, St-Constant, QC, Canada) were used. Animals were housed in ventilated polycarbonate cages and received water and standard laboratory rodent diet (Harlan Teklad, soy-free diet 2020X) *ad libitum*. All experiments were approved by the Animal Care Committee at McGill University, and conformed to the ethical guidelines of the Canadian Council on Animal Care and the guidelines of the Committee for Research and Ethical Issues of the International Association for the Study of Pain published in PAIN, 16 (1983) 109–110. All surgery was performed under isoflurane anesthesia, and all efforts were made to minimize suffering.

### Induction of nerve injury

Neuropathy was induced using the spared nerve injury model. Under deep anesthesia, an incision was made on the lateral surface of the thigh through the muscle, exposing the three terminal branches of the sciatic nerve: the sural, common peroneal and tibial nerves. The common peroneal and the tibial nerves were tightly ligated with 6.0 silk (Ethicon) and sectioned distal to the ligation. The sural nerve was left intact. Sham surgery involved exposing the nerve without damaging it [71,72].

### Behavioral assessment

#### Mechanical sensitivity

Calibrated monofilaments (Stoelting Co., Wood Dale, IL) were applied to the plantar surface of the hindpaw and the 50% threshold to withdraw (grams) was calculated as previously described [73]. The stimulus intensity ranged from 0.008 g to 4 g.

#### Cold sensitivity

A modified version of the acetone drop test [74] was used: total duration of acetone evoked behaviors (flinching, licking or biting) was measured for 1 minute after acetone (~25 μl) was applied to the plantar surface of the hindpaw.

#### Motor function

The accelerating rotarod assay was used (IITC Life Science Inc., Woodland Hills, CA) with the mouse adapter [75]. The task includes a speed ramp from 0 to 30 rpm over 60 s, followed by an additional 240 s at the maximal speed.

### Tissue extraction

Animals were sacrificed 6 months after nerve injury or sham surgery by decapitation following isoflurane anesthesia. Anatomical regions were defined according to the stereotaxic coordinates (rostral–caudal, medial–lateral and dorsal–ventral from bregma) by Paxinos and Franklin [76]. The prefrontal cortex (right and left; +1 to +3, -1 to +1, 0 to −2.5) was extracted, frozen on dry ice and stored at −80 C until use.

### RNA extraction and RNA sequencing

RNA was extracted with Trizol (Invitrogen) according to the manufacturer’s protocol and treated with DNAse. cDNA libraries and RNA sequencing was performed by Genome Quebec on the Illumina Genome Analyzer IIX following Illumina guidelines.

#### Expression validation

3 ug of RNA was used for cDNA conversion using random hexamers (Roche Molecular Biochemicals) according to manufacturer’s guidelines. Expression primers were designed over overlapping exons with Autoprime.de [77] or specific to single exons that were differentially expressed in RNAseq and are shown in Additional file 3: Table S3. All reactions for all subjects (n=6-8 per group) were performed on the LightCycler 480 (Roche) in triplicate and statistical significance was determined as P<0.05 using one-tailed t-tests. Quantitative PCR was amplified with a pre-incubation at 95°C for 10 minutes followed by 45 cycles of [95°C for 10 seconds, 60°C for 10 seconds, 72°C for 10 sec] followed by 10 minutes of 72°C. Expression was measured relative to GAPDH expression, which demonstrated stable unchanged expression from RNAseq results.

### RNAseq analysis

Two animals were sequenced per condition with 36 bp reads with single end sequencing. RNAseq reads were aligned to the genome (mm9) using Top Hat [59,78]. Between 36 and 40 million reads aligned to unique genomic locations. Differential expression was determined from read counts per transcript, exon and 1000 bp genomic partition using Bioconductor [79] package DESeq [80] with default parameter settings. Transcript annotations were obtained from Ensembl version 63.37 (http://www.ensembl.org/). Concordance of read counts per 1000bp genomic partition between pairs of samples ranged between 0.88 to 0.99.

#### Pathway analysis

Ingenuity Software was used to perform whole pathway analysis in the identification of affected networks and their relationship to each other based on the differential expression between SNI and Sham treated mice. Briefly, our data set containing gene identifiers and corresponding expression values was uploaded into the application. Each identifier was mapped to its corresponding object in the Ingenuity® Knowledge Base. Differentially expressed genes, called Network Eligible molecules, were overlaid onto a global molecular network developed from information contained in the Ingenuity Knowledge Base. Networks of Network Eligible Molecules were then algorithmically generated based on their connectivity. Pathways presented were chosen from following candidate gene validation (as indicated with asterisks in Table 2). Right-tailed Fisher’s exact test was used to calculate a p-value determining the probability that each pathway, biological function and/or disease assigned to that data set is due to chance alone.

## Abbreviations

CLCA1: Calcium-activated chloride channel regulator 1; CNS: Central nervous system; GFAP: Glial fibrillary acidic protein; GRIN 1: Glutamate receptor, ionotropic, N-methyl D-aspartate 1; IPA: Ingenuity pathway analysis; KRT20: Keratin 20; LBP: Lipopolysaccharide binding protein; PCR: Polymerase chain reaction; PFC: prefrontal cortex; RNAseq: RNA sequencing; ROBO3: Roundabout, axon guidance receptor, homolog 3; SCN1A: Sodium channel, voltage -gated, type I, alpha subunit; SNI: Spared nerve injury; SYT2: Synaptotagmin II; XLR4B: X-linked lymphocyte-regulated 4B.

## Competing interests

The authors declare that they have no competing interests.

## Authors’ contributions

SA and MT Overall experimental design, collecting the mouse behavioral data, performing RNA data collection and analysis and drafting the manuscript. MM Induction of nerve injury, collection of brain tissue Suderman: Bioinformatic analysis of RNAseq data. LSS Overall design of the experiments, supervision of the experiments and writing of the manuscript. Szyf: Overall design of the experiments, supervision of the experiments and writing of the manuscript. All authors have read and approved the final manuscript.

## Supplementary Material

Additional file 1: Table S1 List of differentially regulated genes as revealed by RNAseq. Click here for file

Additional file 2: Table S2 Ingenuity Pathway Analysis of altered biological functions in Sham vs. SNI PFC. Click here for file

Additional file 3: Table S3 QPCR primer sets used for validation. Click here for file
